# Anthropomorphic AI and Consumer Skepticism: A Behavioral Study of Trust and Adoption in Fragile Economies

**DOI:** 10.3390/bs16040496

**Published:** 2026-03-27

**Authors:** Agnes Caroline Dontina Mackay, Li Zuo, Ibrahim Alusine Kebe

**Affiliations:** 1 School of Economics and Management, Beijing Jiaotong University, Beijing 100044, China; 2 Institute of Public Administration and Management, University of Sierra Leone, Freetown 999127, Sierra Leone

**Keywords:** AI anthropomorphism, trust in AI, perceived social presence, digital skepticism, consumer behavior, fragile economies, behavioral intention

## Abstract

This study examines the psychological mechanisms through which anthropomorphic artificial intelligence (AI) relates to consumer adoption intentions in fragile, low-trust economies. Integrating the Stimulus–Organism–Response framework with the Computers Are Social Actors paradigm, Institutional Trust Theory, and Privacy Calculus Theory, we investigate how human-like AI design shapes cognitive and affective responses within Sierra Leone’s banking sector. Using survey data from 277 banking customers and partial least squares structural equation modeling, we find that AI anthropomorphism exhibits no direct association with adoption intention (β = −0.013, *p* = 0.760). Instead, its influence is entirely indirect—transmitted in parallel through perceived social presence (β = 0.144, 95% CI [0.062, 0.226]) and trust in the AI system (β = 0.139, 95% CI [0.068, 0.210]). Critically, customer skepticism—shaped by institutional fragility—functions as a boundary condition that substantially attenuates both pathways: among highly skeptical users (+1 SD), anthropomorphism’s conditional effect on social presence becomes non-significant (β = 0.098, *p* = 0.124) compared to low-skepticism users (β = 0.412, *p* < 0.001), while its effect on trust is reduced by more than half (β = 0.118 vs. 0.284). These findings identify a critical boundary condition on human-like AI design: in low-trust environments, anthropomorphism operates not as a standalone adoption driver but as a relational amplifier whose efficacy depends on foundational trust and is substantially weakened when skepticism is high. The study challenges universalist assumptions in human–AI interaction research and underscores the need for institutionally sensitive design approaches in fragile economies.

## 1. Introduction

Artificial intelligence (AI) is rapidly transforming financial services worldwide. From virtual assistants that guide loan applications to chatbots that resolve customer queries in real time, AI-driven interfaces are redefining how banks engage with clients (Bhatnagr & Rajesh, 2025). A growing body of research suggests that designing AI systems with human-like qualities such as voice intonation, empathetic language, or even a name can enhance user experience by triggering social responses (Peter et al., 2025; Truong & Chen, 2025). This design strategy, known as AI anthropomorphism, has been shown to increase perceived warmth, reduce psychological distance, and foster trust in high-income contexts (Blut et al., 2021). These findings largely emerge from technologically mature, high-trust societies—raising a critical question: Does anthropomorphism yield similar benefits in fragile, resource-constrained economies where institutional trust is low and digital skepticism is high?

Theoretical advances in human–AI interaction often rely on the Computers Are Social Actors (CASA) paradigm (Nass & Moon, 2000), which posits that users automatically apply social heuristics to machines that exhibit human cues. More recently, scholars have embedded CASA within the broader Stimulus–Organism–Response (S-O-R) framework (Mehrabian & Russell, 1974) to explain how external design features (stimuli) evoke internal cognitive-affective states (organism), which in turn shape behavioral intentions (response) (J. Li et al., 2025; Zhou & Ma, 2025). For instance, recent studies demonstrated that anthropomorphic cues in AI assistants increase perceived social presence—a core organismic state—which subsequently boosts continuance intention (Chae & Kim, 2026).

Similarly, Y. Li et al. (2024) found that robots with human-like appearances increase consumer trust through perceived warmth and competence, with effects varying across cultures and service contexts.

Despite these insights, a significant contextual and theoretical gap persists. Nearly all empirical studies on AI anthropomorphism have been conducted in WEIRD (Western, Educated, Industrialized, Rich, Democratic) societies (Greilich et al., 2025; T. Li et al., 2025) where digital infrastructure is robust, regulatory frameworks for AI are emerging, and citizens generally exhibit higher baseline trust in institutions. In stark contrast, Sub-Saharan Africa—home to some of the world’s most dynamic yet fragile digital finance ecosystems—remains conspicuously absent from this literature (Ofori-Okyere & Edghiem, 2026). While pioneering studies have examined Internet banking acceptance in South Africa (Maduku, 2016), research on anthropomorphic AI in banking across the region remains limited. This omission is problematic for two reasons. First, it risks theoretical overgeneralization: mechanisms that work in Zurich may fail in Freetown due to differences in digital literacy, cultural norms around authority, or historical experiences with institutional betrayal (Donner, 2015). Second, it overlooks a critical policy and design frontier: as financial institutions in Africa seek to leverage AI to expand financial inclusion—a promising pathway outlined by Akanfe et al. (2025)—they urgently need evidence-based guidance on how to design these systems to build trust and resonate, rather than alienate, users in low-trust, fragile environments like Sierra Leone.

Nowhere is this more urgent than in Sierra Leone, a nation navigating the complex legacy of civil conflict and public health crises, which represents a critical case study of digital transformation within a low-trust, post-crisis environment. The country’s banking sector is undergoing rapid digitization, with financial institutions piloting AI chatbots to reach unbanked populations (Kamara & Oppong, 2025; Kebe et al., 2024); however, customer adoption remains sluggish. Preliminary fieldwork suggests that while some users find human-like AI “friendly,” others express deep skepticism—questioning whether a machine can understand their needs or protect their data in a system with weak oversight. This tension points to a pivotal but unexamined boundary condition: individual skepticism toward AI, shaped by socio-institutional fragility, may fundamentally alter how anthropomorphic stimuli are processed internally

To address this gap, we ask:

1. *How does AI anthropomorphism influence customers’ intention to adopt AI-driven banking services?*
2. *To what extent are the effects of AI anthropomorphism influenced by perceived social presence and trust in the AI system?*
3. *How does customer skepticism moderate the psychological pathways linking anthropomorphism to adoption intention?*

Guided by the S-O-R framework, we conceptualize AI anthropomorphism as the stimulus (S), with perceived social presence (an affective-relational state) and trust in the AI system (a cognitive-evaluative state) operating as parallel organismic dimensions within the ‘O’ component. These concurrent psychological states jointly translate anthropomorphic stimuli into adoption intention (R), with skepticism acting as a dispositional moderator of both pathways. This parallel architecture reflects users’ adaptive dual-channel processing in fragile economies—simultaneously appraising relational warmth and institutional safety when evaluating AI systems.

However, this study makes three key contributions. First, it challenges the presumed universality of established human-AI interaction theories by demonstrating that anthropomorphism exerts no direct effect on adoption in fragile economies; its influence is entirely indirect, operating through perceived social presence and trust. This finding reveals that human-like design is not a standalone driver of adoption but a relational amplifier whose efficacy is contingent on foundational trust conditions—a critical boundary condition on CASA. Second, it introduces and empirically validates individual skepticism as a critical boundary condition, revealing a “trust duality” where human-like design can backfire in low-trust, high-skepticism environments. Third, it provides actionable, context-sensitive guidance for stakeholders, shifting the focus from design-centric to trust-centric AI deployment strategies that prioritize institutional accountability and user empowerment in the Global South.

## 2. Literature Review and Hypotheses

### 2.1. Theoretical Framework

This study is grounded in the Stimulus–Organism–Response (S-O-R) framework (Mehrabian & Russell, 1974), which posits that external cues evoke internal psychological states that shape behavioral outcomes. Critically, the organism (‘O’) comprises multiple concurrent states—not sequential stages. Mehrabian and Russell (1974) originally conceptualized pleasure, arousal, and dominance as simultaneous dimensions activated by stimuli.

Extending this logic, we theorize that AI anthropomorphism activates two parallel organismic dimensions in fragile economies: (1) an affective-relational channel (perceived social presence) reflecting interpersonal warmth and (2) a cognitive-institutional channel (trust) reflecting risk appraisal (Gefen & Straub, 2004; Mcknight et al., 2011). We propose this parallel architecture as contextually rational in Sierra Leone: when institutional safeguards are weak, users may not afford sequential processing that prioritizes relational connection before safety evaluation. Instead, we suggest they engage in dual-channel vigilance—simultaneously appraising whether the AI ‘feels human’ and whether it ‘can be trusted.’

We integrate three theories to explain this architecture. CASA (Nass & Moon, 2000) explains why anthropomorphism functions as a potent stimulus that triggers social attributions. Institutional Trust Theory (Mcknight et al., 2011) clarifies how trust emerges concurrently with social presence as users rely on relational signals as proxies for benevolence in low-regulation environments. Privacy Calculus Theory (Dinev & Hart, 2006) positions skepticism as a boundary condition that simultaneously attenuates both pathways when users perceive anthropomorphic cues as manipulative rather than benevolent. Together, these theories position anthropomorphism as a context-dependent stimulus whose efficacy hinges on institutional fragility and user skepticism within a parallel-processing S-O-R model.

Collectively, these frameworks operate at distinct analytical levels. The S-O-R framework provides the overarching structural architecture, mapping the causal sequence from design features to behavioral outcomes without prescribing the specific psychological content. CASA specifies the micro-psychological mechanism, explaining why anthropomorphic stimuli automatically trigger social heuristics rather than technical evaluations. Institutional Trust Theory contextualizes the organismic state, clarifying why trust must operate as a parallel pathway to social presence in fragile economies where relational signals substitute for weak regulatory safeguards. Finally, Privacy Calculus Theory defines the boundary condition, explaining when the process fails—specifically, when skeptical users recalibrate the risk–benefit analysis to view human-like cues as manipulative rather than benevolent. Together, this integrated lens moves beyond a generic application of S–O-R to offer a contextually situated explanation of AI adoption in low-trust environments.

### 2.2. Hypotheses Development and Conceptual Framework

#### 2.2.1. AI Anthropomorphism as a Stimulus in Digital Service Environments

In human–AI interaction research, anthropomorphism, regarded as the attribution of human-like qualities such as voice, emotion, name, or intentionality to non-human agents, has emerged as a powerful design strategy for enhancing user engagement (Keating et al., 2025). Grounded in CASA, which predicts that human-like cues automatically trigger social responses, we hypothesize that AI anthropomorphism will positively influence both perceived social presence (the affective-relational outcome of social cue processing) and trust (the cognitive-evaluative outcome, as social heuristics also signal benevolence). Within the S-O-R framework, AI anthropomorphism functions as an external stimulus that elicits internal cognitive and affective states—the organism—which subsequently shape behavioral outcomes—the response (Pan et al., 2024). Empirical evidence consistently demonstrates that anthropomorphic cues activate social heuristics, leading users to perceive AI systems as warmer, more empathetic, and more responsive (Greilich et al., 2025; J. Li et al., 2025). In banking contexts where trust, personalization, and emotional safety are paramount, such design features have been shown to enhance Perceived Social Presence and mitigate anxiety associated with digital financial transactions (Chang et al., 2025). However, a primary mechanism through which anthropomorphism operates is perceived social presence—defined as the extent to which users experience interaction with a sentient, responsive entity rather than a passive technological tool (Tsekouras et al., 2024). When AI systems display human-like attributes, users are more likely to interpret the interaction as socially meaningful, thereby strengthening feelings of co-presence and interpersonal connection, and this effect is especially pronounced in service domains requiring emotional resonance (Y. Li et al., 2024). Accordingly, we hypothesize:

**H1.**  *AI anthropomorphism positively influences perceived social presence.*

Beyond its influence on social presence, anthropomorphism may also have a trust-building effect. Trust in technology is often formed rapidly through surface-level cues, especially in low-involvement or high-uncertainty contexts. Anthropomorphic features such as a friendly tone, empathetic phrasing, or a personalized name serve as trust heuristics, signaling benevolence and competence without requiring users to engage in complex evaluations of system logic, data security, or algorithmic transparency (Calhoun et al., 2019). In banking, where perceived integrity is critical, these cues can directly elevate trust, even prior to the full development of social presence. A systematic literature review of 84 studies found that anthropomorphic chatbots generally produce positive outcomes, with humanlike communication styles and emotional characteristics enhancing trust, empathy, and social presence, though overly humanlike features can trigger privacy concerns and AI anxiety (Greilich et al., 2025). Similarly, Z. Wang et al. (2026) found that human-likeness in AI financial advisors positively affects adoption intention through consumer technology vulnerability, though this effect is diminished by higher self-efficacy and consumer innovativeness.

In Sierra Leone—a context marked by limited digital literacy and underdeveloped mental models of AI—users may rely heavily on such heuristic cues to assess reliability. Thus, anthropomorphism may function as a cognitive proxy for trustworthiness, amplifying its direct impact. We therefore propose:

**H2.**  *AI anthropomorphism positively influences trust in the AI system.*

Furthermore, anthropomorphism can also shape behavioral intention through affective and normative mechanisms, independent of trust or social presence. Users may adopt AI-driven banking services simply because they find the interface likable, engaging, or culturally resonant—responses triggered automatically by human-like design (Bhatnagr et al., 2024). Research on human–machine interaction reveals that interface design and repeated exposure significantly influence user preferences and comfort. Tsekouras et al. (2024) demonstrated that more anthropomorphic conversational interfaces lead to increased interaction enjoyment and social presence, resulting in more positive user responses. In resource-constrained settings like Sierra Leone, where interpersonal warmth and relational familiarity are deeply embedded in service expectations, an AI that “speaks like a neighbor” may be embraced not because it is technically understood or fully trusted, but because it feels familiar, respectful, and socially appropriate. This aligns with von Schenk et al. (2025) which found that people form beliefs about machine interactions in self-serving ways, indicating that preferences can develop without conscious evaluation. Given the cultural and institutional context of Sierra Leone’s banking sector, this study proposes a similar direct association between anthropomorphic design and adoption behavior.

**H3.**  *AI anthropomorphism positively influences intention to adopt AI-driven banking services.*

#### 2.2.2. The Role of Perceived Social Presence

Perceived social presence—the subjective sense that one is interacting with a sentient, responsive entity rather than an impersonal machine—serves as a critical psychological mechanism in human–AI interaction (Gefen & Straub, 2004). Drawing on S-O-R, perceived social presence functions as a core organismic state that translates external design cues into behavioral responses. From a CASA perspective, this state is activated precisely because users apply social heuristics to anthropomorphic stimuli. In service contexts where relational quality shapes user experience, social presence bridges the gap between technological functionality and human-centered engagement (Y. Li et al., 2024). Kandampully et al. (2023) emphasize that managers must understand consumers’ emphasis on social presence alongside technological personalization, aesthetics, functionality, and interactivity while participating in value cocreation. However, having a strong sense of social presence enhances user comfort, reduces perceived transactional risk, and fosters emotional connection—all of which directly promote adoption intentions. When users feel accompanied by an AI agent, they are more likely to perceive the interaction as supportive, intuitive, socially valid and outperforming other activities like watching videos (De Freitas et al., 2025). This is especially consequential in banking, a domain characterized by high involvement, emotional sensitivity, and institutional vulnerability. Empirical studies confirm that higher social presence increases willingness to disclose financial information, follow AI advice, and continue using digital services (Bhatnagr et al., 2024), consequently, Riedel et al. (2022) demonstrated that consumers experience lower positive emotions, specifically affection, when receiving financial advice from AI compared to humans, with affection and trust serving as serial mediators affecting word-of-mouth and brand attitudes. Considering Sierra Leone’s context, where face-to-face banking remains culturally normative and digital interfaces may feel alienating, social presence can mitigate resistance by simulating interpersonal rapport. Users who perceive the AI as present and attentive are more likely to view it as a legitimate service partner—thereby increasing their intention to adopt. Thus, we propose:

**H4.**  *Perceived social presence positively influences intention to adopt AI-driven banking services.*

#### 2.2.3. The Role of Trust in AI

Trust—the willingness to be vulnerable to another party based on positive expectations of their behavior (Mayer et al., 1995)—is a cornerstone of technology adoption, especially in high-stakes domains such as financial services. Informed by Privacy Calculus Theory, trust operates as a risk-mitigating organismic state: in high-stakes banking contexts, users must believe the system will protect their data before adopting it. Within the S-O-R architecture, trust thus represents the cognitive-evaluative dimension of the organism. In the context of AI-driven banking, trust in the AI system reflects users’ beliefs that the technology is competent, benevolent, and operates with integrity (Königstorfer & Thalmann, 2020). Within the S-O-R framework, trust functions as a critical organismic state that translates cognitive and affective appraisals into behavioral intentions.

In digital finance, where decisions involve personal data, monetary risk, and long-term consequences, trust serves as a primary antecedent of user engagement. When customers believe an AI system will protect their information, provide accurate advice, and act in their best interest, they are significantly more likely to adopt and rely on it (Z. Wang et al., 2026). This relationship is robust across contexts as evidence reveals that trust entities show comparable paths to intention to use, with trust in technology being more important than trust in the provider when both are considered (Kuen et al., 2023). The historical institutional fragility and limited digital regulation in Sierra Leone elevate the importance of trust in AI adoption. Here, user hesitation frequently originates in practical concerns such as data security and fairness, rather than aversion to technology. Establishing trust reduces these perceived risks and facilitates adoption. Accordingly, we hypothesize:

**H5.**  *Trust in the AI system positively influences intention to adopt AI-driven banking services.*

#### 2.2.4. The Mediating Role of Perceived Social Presence and Trust in AI System Outcomes

Beyond its ability to influence intention to adopt AI-driven banking services, perceived social presence also serves as a key factor in the pathway from AI anthropomorphism to behavioral intention. Anthropomorphic design through voice, empathy, or conversational style does not influence behavior in isolation; rather, it activates the perception of social presence, which is associated with adoption (Cheng et al., 2022). Similarly, healthcare conversational agents demonstrate that anthropomorphism serves as one of multiple factors influencing acceptability, acceptance, and adoption among both patients and professionals. Meta-analytic evidence supports this mediation: Blut et al. (2021) found that social presence consistently mediates the effect of anthropomorphism on user satisfaction and behavioral outcomes across service robots and virtual agents. Similarly, Bhatnagr et al. (2024) demonstrated that in digital banking, anthropomorphism and perceived intelligence predict interaction quality, which significantly impacts expectation confirmation and continuous intention to use AI-enabled services. Given that users in fragile economies like Sierra Leone may rely more on relational heuristics than technical assessments when evaluating AI, perceived social engagement is likely to be a decisive factor in shaping behavioral intention. Consequently, we hypothesize that perceived social presence operates as an independent mediating pathway through which AI anthropomorphism is associated with adoption intention—reflecting the affective-relational channel activated by human-like design cues.

**H6.**  *Perceived social presence mediates the relationship between AI anthropomorphism and intention to adopt AI-driven banking services.*

Further, Trust in the AI system operates as a distinct, independent mediating pathway through which AI anthropomorphism influences adoption intention—reflecting the cognitive-institutional channel through which users evaluate system benevolence and integrity. Critically, this pathway operates in parallel with (not subsequent to) the social presence pathway, as both represent distinct organismic dimensions within the S-O-R framework. While anthropomorphic cues (e.g., empathetic language, human-like voice) may initially enhance appeal, their ultimate value lies in fostering perceived trustworthiness. Human-like attributes signal benevolence and social alignment, leading users to infer that the AI “cares” about their welfare—a key dimension of trust in automated systems (Yu et al., 2025). Bhatnagr et al. (2024) demonstrated that in conversational banking agents, anthropomorphism increased adoption intention primarily through enhanced trust, even after controlling for usability and social presence. Similarly, Z. Wang et al. (2026) found that human-likeness in AI financial advisors positively affects adoption intention through reduced consumer technology vulnerability, though this effect is moderated by individual traits like self-efficacy and consumer innovativeness. Curtis et al. (2021) also found that empathy, relational behaviors, and realistic human-like avatars with medical attire enhance user experience with virtual health assistants, suggesting that carefully designed anthropomorphic features can improve engagement with AI-based advisory services. These findings align with institutional trust theory, which posits that in environments with weak formal safeguards, users rely on relational signals like human-likeness to assess reliability (Mcknight et al., 2011).

Considering Sierra Leone’s relational culture, where interpersonal trust often compensates for weak institutional confidence, an AI that feels human may be perceived as more accountable and less likely to exploit users. Consequently, we hypothesize:

**H7.**  *Trust in the AI system mediates the relationship between AI anthropomorphism and intention to adopt AI-driven banking services.*

#### 2.2.5. The Moderating Role of Skepticism

While AI anthropomorphism can enhance user perceptions through social and trust-based mechanisms, its effectiveness is not universal. Institutional Trust Theory suggests that in weak regulatory environments, trust is not automatically granted but filtered through skepticism born of institutional experience. Privacy Calculus Theory further predicts that skeptical users will perceive greater risks than benefits from anthropomorphic cues. Thus, skepticism moderates both pathways, attenuating the translation of anthropomorphism into social presence and trust. Individual differences like skepticism toward AI can significantly weaken these effects. Skepticism, defined as a dispositional tendency to doubt the motives, competence, or safety of artificial intelligence systems (da Costa Filho & da Costa Hernandez, 2025), functions as a critical boundary condition in the S-O-R chain. It shapes how users interpret and respond to anthropomorphic stimuli, especially with people prone to underestimating AI potential due to exponential growth bias and motivated reasoning (Meikle & Bonner, 2024). Consistent with S-O-R, both perceived social presence and trust function as mediating organismic states. CASA predicts that anthropomorphism activates these states, while Privacy Calculus Theory explains why trust—specifically—carries the cognitive risk appraisal necessary for adoption in banking contexts. Users high in skepticism are more likely to engage in counter-arguing or threat appraisal when exposed to human-like AI, interpreting warmth as manipulation and empathy as deception (Peter et al., 2025). This cognitive resistance weakens the psychological pathways through which anthropomorphism typically operates.

Perceived social presence arises when users accept anthropomorphic cues at face value, allowing themselves to experience the interaction as socially meaningful. Skeptical individuals may discount or reinterpret these cues as artificial or strategic, thereby blocking the sense of co-presence. For example, a friendly greeting from an AI chatbot might be perceived by a low-skepticism user as warm, but by a high-skepticism user as scripted or deceptive. This aligns with privacy calculus theory, which posits that users weigh perceived benefits against risks and skepticism heightens risk perception (Dinev & Hart, 2006). Empirical support comes from the mHealth domain, von Kalckreuth and Feufel (2023) found that intention to use mHealth apps is driven by perceived benefits, trust, and social norms, with attitude to privacy having a large inhibiting effect on perceived benefits. This skepticism is not unique to Sierra Leone. In Nigeria, (Okeke, 2025), documents similar distrust toward AI chatbots, citing data security concerns and linguistic mismatches as key barriers. Despite sophisticated deployments like First Bank’s Ada, adoption remains constrained by ‘trust deficits rooted in institutional experience’—directly paralleling our conceptualization of skepticism as institutionally shaped rather than merely technological. We therefore anticipate that higher skepticism will weaken the positive relationship between anthropomorphism and social presence.

**H8.**  *Skepticism negatively moderates the positive relationship between AI anthropomorphism and perceived social presence, such that the relationship is weaker when skepticism is high.*

Similarly, skepticism disrupts the formation of trust in response to anthropomorphic design. Trust requires vulnerability, yet skeptical users are predisposed to attribute negative intent to AI systems, regardless of surface-level friendliness (Mcknight et al., 2011). A human-like voice or empathetic phrasing may even backfire, triggering suspicion that the AI is trying too hard to gain compliance. Brandizzi (2023) emphasizes that developing human-like AI communication requires understanding human-AI trust dynamics, as misalignment between AI language use and human reasoning can lead to unexpected behaviors. Also, in financial advising, anthropomorphism increased trust only among users with low AI skepticism; for highly skeptical users, the same cues had no effect or even reduced trust (Z. Wang et al., 2026). This reflects a motivated reasoning process: when users doubt the legitimacy of a technology, they scrutinize its signals more critically, rendering heuristic cues ineffective. Within Sierra Leone’s banking sector, historical institutional fragility fuels public distrust, which in turn heightens skepticism toward AI systems. Consequently, anthropomorphic design cannot fully overcome underlying doubts regarding data ethics, algorithmic bias, or organizational intent. Instead, its ability to foster trust is contingent upon pre-existing skepticism. Hence, we hypothesize:

**H9.**  *Skepticism negatively moderates the positive relationship between AI anthropomorphism and trust in the AI system, such that the relationship is weaker when skepticism is high.*

### 2.3. Synthesis of Hypotheses

**Figure 1:**
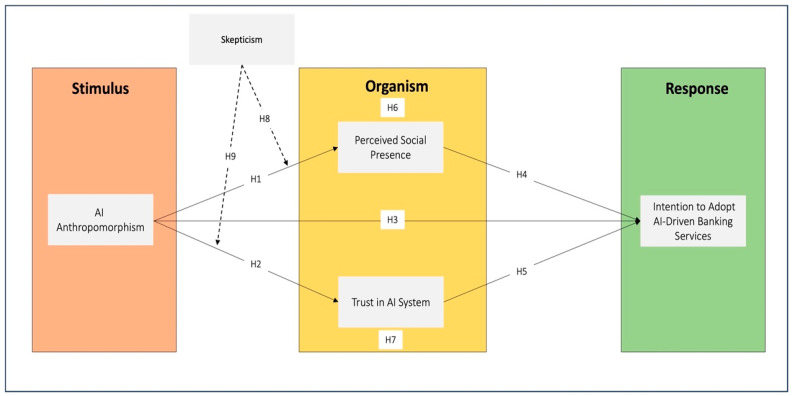
Conceptual Framework. Note: Solid arrows represent direct hypothesized effects. Dashed arrows represent moderating effects.

The model framework (see Figure 1) integrates the CASA paradigm, Institutional Trust Theory, and Privacy Calculus Theory within the S-O-R framework to explain AI adoption in Sierra Leone’s banking sector. CASA establishes dual pathways: AI anthropomorphism (stimulus) simultaneously activates two parallel organismic dimensions—perceived social presence (affective-relational) and trust (cognitive-institutional)—which jointly drive adoption intention (response). This parallel architecture is not a methodological artifact but a contextually rational response to institutional fragility: in low-trust environments, users cannot afford sequential processing that prioritizes warmth before safety. Instead, they engage in dual-channel appraisal—simultaneously evaluating relational connection and institutional risk.

However, in contexts of institutional fragility and weak data governance, skepticism, shaped by Privacy Calculus and Institutional Trust Theory, acts as a contextual filter. It determines whether human-like cues are seen as benevolent or manipulative. The study proposes a trust duality: anthropomorphism strengthens adoption when skepticism is low but may backfire when skepticism is high. This integrated view captures not only how AI design influences behavior, but when it succeeds, thereby highlighting that effective AI deployment in fragile economies requires more than good design; it demands alignment with local socio-institutional realities.

## 3. Methodology

### 3.1. Research Strategy and Design

This study adopts a quantitative, cross-sectional survey design to test a moderated mediation model grounded in the S-O-R framework. The design is appropriate for examining complex psychological mechanisms such as the roles of perceived social presence, trust, and skepticism in shaping behavioral intentions toward AI-driven banking services (Podsakoff et al., 2003). Given the novelty of AI interfaces in Sierra Leone’s financial sector, a survey approach enables systematic measurement of latent constructs that cannot be directly observed but are reliably captured through validated self-report scales (Hair et al., 2021).

### 3.2. Population and Sampling Procedure

The target population consists of adult banking customers (aged ≥ 18) in Sierra Leone who have either used, been exposed to, or at least heard about AI-driven banking services—such as chatbots, virtual assistants, or AI-powered SMS alerts—within the past 12 months. Given the nascent stage of AI adoption in the country’s financial sector, we included individuals with even minimal awareness (e.g., having seen a demonstration or received promotional information) to ensure meaningful cognitive engagement with the survey constructs.

Participants were selected using a stratified random sampling approach across four major urban areas representing all four administrative regions of Sierra Leone: Freetown (Western Area), Makeni (Northern Province), Bo (Southern Province), and Kenema (Eastern Province). These locations collectively account for the majority of formal banking activity in the country (Ministry of Finance—Government of Sierra Leone, 2023). Within each city, we randomly selected branches from a mix of domestic and foreign-based commercial banks. At each branch, research assistants approached customers during non-peak hours and screened them for eligibility using a brief verbal questionnaire. Eligible and consenting individuals were then invited to participate in the survey.

Data were collected between August and December 2025 using via KoboToolbox, a secure digital data collection platform. This method accommodated varying literacy levels by allowing trained enumerators to read questions aloud in English or Krio while recording responses.

To determine the minimum sample requirements, an a priori power analysis was conducted using G*Power 3.1 for multiple regression (α = 0.05, power = 0.95, medium effect size f^2^ = 0.15). This analysis indicated a minimum of 276 respondents to detect the direct effects among six predictors. To account for potential attrition, we targeted 350 eligible participants, ultimately securing 277 complete responses (89.4% completion rate).

While this power analysis establishes a baseline for detecting main effects, moderated mediation models introduce interaction terms and indirect effects, which typically exhibit smaller effect sizes and require scrutiny of model complexity. To ensure the sample was adequate for the proposed analytical strategy, we evaluated it against Structural Equation Modeling (SEM) criteria. The final sample of 277 satisfies three critical requirements: (1) it exceeds the N ≥ 200 threshold recommended by Hair et al. (2021). for PLS-SEM to obtain stable path estimates with medium effects; (2) it maintains a robust 9.2:1 ratio of respondents to estimated parameters (30 parameters), which exceeds the 5:1 minimum recommended for SEM and mitigates the risk of overfitting; and (3) following recommendations by (Aguinis et al., 2013) for detecting interaction effects, the sample provides sufficient statistical power to detect moderation effects of medium-to-large magnitude. Therefore, the sample is deemed adequate for the complexity of the proposed moderated mediation model.

### 3.3. Measurement Scales

All constructs were measured using 5-point Likert scales (1 = strongly disagree, 5 = strongly agree) and adapted from validated instruments (see Appendix A). AI Anthropomorphism was defined as the perception that an AI-driven banking interface exhibits human-like qualities such as voice, empathy, or intentionality. A 5-item scale was adapted from (Bartneck et al., 2009). Perceived Social Presence was measured with a 4-item scale from (Gefen & Straub, 2004), capturing the sense of interacting with a sentient entity. Trust in the AI System employed a 5-item scale adapted from (Chowdhury et al., 2022), reflecting competence, benevolence, and integrity. Intention to Adopt AI-Driven Banking Services used a 5-item scale from (Liu et al., 2022). Skepticism toward AI was measured with a 3-item scale adapted from (Zhang et al., 2016) and contextualized for financial services in low-trust environments.

All scales underwent a three-stage cultural adaptation process: (1) expert panel review for linguistic/relational/technological grounding; (2) cognitive pre-testing (N = 24) with think-aloud protocols to identify misinterpretations; and (3) pilot testing (*n* = 30) with EFA validation; For example, the anthropomorphism item ‘has a personality’ was revised to ‘communicates in a friendly, human-like way’ after cognitive interviews revealed spiritual misinterpretations; trust items were reframed to acknowledge Sierra Leone’s weak regulatory environment (e.g., ‘even though data laws are weak here, this AI tries to protect my information’). All items were administered in Krio with enumerator clarification protocols to ensure construct equivalence (Brislin, 1970).

### 3.4. Data Collection and Analysis

Anonymous surveys were administered to customers at commercial banks across four urban centers in Sierra Leone: Freetown, Makeni, Bo, and Kenema. Eligibility required participants to be adults (≥18 years) with prior exposure to AI-driven banking services (e.g., chatbots, virtual assistants, or AI-powered alerts) in the preceding 12 months. To mitigate common method bias and social desirability, item order was randomized, and trained research assistants conducted the surveys. A pilot test (*n* = 30) confirmed the clarity and cultural appropriateness of the instrument in both English and Krio, with an average completion time of 12 min. Missing data were minimal (<1.5% per item) and handled using series mean imputation after confirming data were missing completely at random (MCAR) via Little’s test: χ^2^ = 31.24, *p* = 0.132.

Data were analysed using Partial Least Squares Structural Equation Modeling (PLS-SEM) in SmartPLS 4.0. This approach was selected for three methodologically grounded reasons aligned with the study’s aims. First, the research extends rather than tests established theory—applying the S-O-R and CASA frameworks to Sierra Leone’s fragile banking context, where measurement models developed in WEIRD settings may not transfer cleanly. PLS-SEM’s component-based estimation accommodates such uncertainty, whereas covariance-based SEM assumes perfect a priori measurement specification. Second, the study’s predictive orientation—centred on whether and how AI adoption drives usage intention—prioritises out-of-sample prediction (maximising R^2^ and Q^2^ predict), which PLS-SEM optimises. Third, the field data exhibited non-normality (e.g., skewness = 1.82, kurtosis = 3.47 for skepticism); PLS-SEM with bootstrapping is robust to non-normal distributions without requiring large samples (N > 500). Collectively, these considerations render PLS-SEM methodologically appropriate for technology adoption research in Global South contexts, where theory extension, predictive focus, and measurement flexibility are paramount.

The measurement model was evaluated for reliability (Cronbach’s α > 0.80; composite reliability ρₐ > 0.85), convergent validity (AVE > 0.50), and discriminant validity (HTMT < 0.85; Fornell–Larcker criterion satisfied). The structural model was estimated using 5000 bootstrap subsamples to generate bias-corrected 95% confidence intervals for direct, indirect (mediation), and conditional (moderated mediation) effects. Simple slopes were plotted to visualize interaction effects at ±1 SD of skepticism. Model quality was assessed using R^2^ (variance explained), Cohen’s f^2^ (effect size), Stone–Geisser’s Q^2^ (predictive relevance via blindfolding, omission distance = 7), and SRMR (<0.08, indicating good overall fit).

Eight control variables were included to account for demographic and contextual heterogeneity: age, gender, education, income, prior AI experience, bank type, smartphone proficiency, and region. Controls were selected based on (a) theoretical relevance to technology adoption in fragile economies (Venkatesh et al., 2003; Donner, 2015) and (b) significant bivariate correlations (*p* < 0.05) with at least one endogenous construct in preliminary analysis.

## 4. Results

### 4.1. Respondents’ Descriptive Analysis

The sample (N = 277) comprised 57.8% female respondents, predominantly aged 26–45 years (71.5%). Educational attainment was high (64.6% bachelor’s degree or higher). All owned smartphones—an eligibility criterion ensuring engagement with the AI stimulus. Most had prior AI banking exposure (68.2% users; 31.8% aware non-users). Foreign commercial banks were preferred (78.3%), consistent with institutional void theory (North, 1990). Geographically, 54.2% resided in Freetown, with 45.8% distributed across three provincial capitals (Makeni, Bo, Kenema), providing urban–provincial coverage representative of Sierra Leone’s digital banking landscape (see Table 1).

### 4.2. Assessment of Measurement Model

The measurement model was evaluated for reliability, convergent validity, and discriminant validity using guidelines for Partial Least Squares Structural Equation Modeling (PLS-SEM) (Hair et al., 2021). As shown in Table 2, all constructs demonstrate strong internal consistency and item coherence. Composite reliability (CR) values range from 0.774 (Skepticism) to 0.904 (Intention to Adopt), exceeding the recommended threshold of 0.70. Cronbach’s alpha (CA) values similarly exceed 0.70 for all constructs, confirming scale reliability (see Table 2). Convergent validity is supported by factor loadings and average variance extracted (AVE). All indicator loadings are above 0.70 (ranging from 0.721 to 0.906), and AVE values exceed the 0.50 benchmark, with the lowest at 0.608 (AI Anthropomorphism) and the highest at 0.713 (Intention to Adopt). This indicates that each construct explains more than 50% of the variance in its associated items (see Table 2). Discriminant validity was assessed using two criteria. First, the Fornell–Larcker criterion (Table 3) shows that the square root of each construct’s AVE (diagonal values) exceeds its correlations with all other constructs. Second, the Heterotrait–Monotrait ratio (HTMT) (Table 4) confirms discriminant validity, as all HTMT values are below the conservative threshold of 0.85. Collectively, these results confirm that the measurement model exhibits strong reliability, convergent validity, and discriminant validity, satisfying prerequisites for robust structural model estimation.

#### 4.2.1. Structural Model Predictive Power

The structural model demonstrates strong explanatory and predictive capability (Table 5). The endogenous constructs exhibit substantial variance explained (R^2^): INT achieves an R^2^ of 0.621 (adjusted R^2^ = 0.616), indicating that 62.1% of its variance is accounted for by AIA, PSP, and TAI. Similarly, PSP (R^2^ = 0.623) and TAI (R^2^ = 0.515) are robustly predicted by AIA, reflecting theoretically grounded linkages. Predictive relevance was confirmed via Stone–Geisser’s Q^2^ predict (blindfolding, omission distance = 7), with all constructs exceeding the threshold of zero (INT: 0.490; PSP: 0.581; TAI: 0.497), affirming meaningful out-of-sample predictive power (Hair et al., 2021). Prediction errors remain moderate (RMSE ≤ 0.729; MAE ≤ 0.586), consistent with behavioral modeling in resource-constrained contexts. Effect size analysis (f^2^) further reveals that AIA exerts a medium–large influence on PSP (f^2^ = 0.224) and a small–medium influence on TAI (f^2^ = 0.135), while trust demonstrates the strongest proximal effect on adoption intention (f^2^ = 0.214) relative to social presence (f^2^ = 0.173). Critically, the negligible effect of AIA on INT (f^2^ = 0.000) confirms full mediation—anthropomorphism is associated with adoption exclusively through parallel psychological pathways, with no direct behavioral effect. Collectively, these metrics validate a theoretically coherent and contextually grounded model of AI adoption in Sierra Leone’s banking environment.

#### 4.2.2. Common Method Bias (CMB)

To address the potential threat of common method bias inherent in self-reported, single-source data, we implemented a combination of procedural remedies and statistical diagnostics in line with contemporary recommendations (Podsakoff et al., 2012). Procedurally, we (1) psychologically and procedurally separated predictor and criterion variables within the same survey session through item randomization, insertion of neutral filler items, and varied question framing to reduce recall consistency and acquiescence bias; (2) guaranteed respondent anonymity to reduce evaluation apprehension; (3) incorporated reverse-coded items to mitigate acquiescence bias; and (4) employed neutrally worded items to minimize social desirability. Statistically, we conducted three complementary assessments. First, Harman’s single-factor test revealed that a single unrotated factor accounted for 47% of the total variance, falling below the conventional 50% threshold. Second, we applied the marker variable technique using “smartphone satisfaction” as a theoretically unrelated marker; the average correlation change after partialling out the marker covariance was minimal (Δr = 0.052), below the 10% threshold indicative of substantial bias. Third, all construct correlations remained below 0.85, and all inner-model variance inflation factors (VIFs) were comfortably under the conservative cutoff of 3.3 (see Table 2), suggesting that structural estimates are not distorted by collinearity arising from CMB. While common method bias cannot be entirely ruled out in cross-sectional designs, this triangulated approach suggests it is unlikely to substantively influence the reported relationships. We acknowledge this limitation and temper any causal inferences accordingly.

### 4.3. Hypothesis Results

For the study’s hypothesized model, Table 6, Table 7 and Table 8 display the estimated PLS-SEM path coefficients and hypothesis test results. A bootstrapping procedure with 5000 resamples was used to assess all direct, indirect, and moderated effects. This approach was selected for its robustness in generating bias-corrected confidence intervals and accurate significance estimates, particularly for testing mediation (H6 & H7) and moderation (H8 & H9).

#### 4.3.1. Direct Effects

As shown in Table 6, AI anthropomorphism significantly increases perceived social presence (β = 0.354, *p* < 0.001) and trust (β = 0.312, *p* < 0.001), supporting H1 and H2. However, it has no direct effect on adoption intention (β = −0.013, *p* = 0.760). This non-significant direct effect, while leading to rejection of H3, is itself theoretically meaningful: it demonstrates that anthropomorphism does not influence adoption directly but operates entirely through internal psychological states. Combined with the significant indirect effects reported below, this is consistent with full mediation—a finding that challenges universalist assumptions about anthropomorphic design. Both perceived social presence (β = 0.406, *p* < 0.001) and trust (β = 0.445, *p* < 0.001) significantly predict adoption intention, supporting H4 and H5.

#### 4.3.2. Mediation Effects

Both mediation hypotheses are supported (Table 7). Perceived Social Presence (PSP) and Trust in the AI System (TAI) operate as parallel mediators, transmitting the influence of AI anthropomorphism (AIA) to adoption intention (INT) through two concurrent psychological pathways. The specific indirect effects are positive and statistically significant: AIA → PSP → INT (β = 0.144, 95% CI [0.062, 0.226], *p* = 0.001) and AIA → TAI → INT (β = 0.139, 95% CI [0.068, 0.210], *p* < 0.001). Critically, the direct effect of AIA on INT is non-significant (β = −0.013, *p* = 0.760), indicating that anthropomorphism influences adoption exclusively through these internal psychological states—with no residual direct pathway.

#### 4.3.3. Moderation Effects

**Figure 2:**
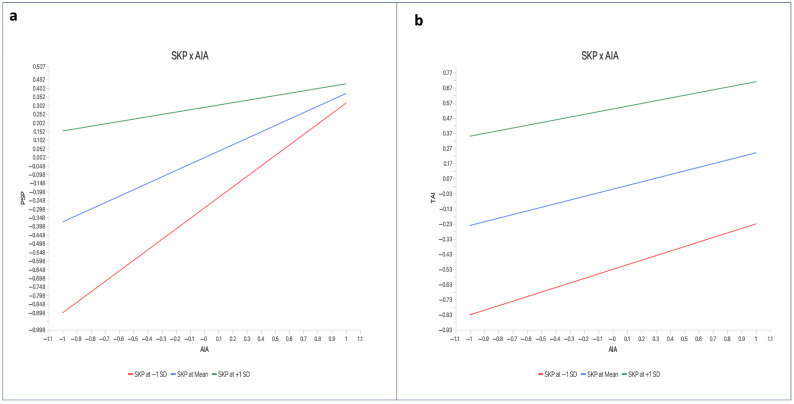
Simple slope analysis of (**a**) SKP’s negative moderation on AIA-PSP relationship, and (**b**) SKP’s negative moderation of AIA-TAI relationship.

As shown in Table 8, skepticism significantly moderates both pathways. The interaction between skepticism and AI anthropomorphism negatively predicts Perceived Social Presence (β = −0.268, *p* < 0.001) and trust in AI (β = −0.090, *p* = 0.001), supporting H8 and H9. Simple slope analyses (Figure 2) confirm that among low-skepticism users, AI anthropomorphism strongly predicts perceived social presence (β = 0.412, *p* < 0.001) and trust (β = 0.284, *p* < 0.001); among high-skepticism users, these effects are markedly attenuated—perceived social presence becomes non-significant (β = 0.098, *p* = 0.124), and trust is more than halved (β = 0.118, *p* = 0.042). In fragile economies like Sierra Leone, where institutional distrust fuels skepticism, these findings underscore that AI deployment alone is insufficient; it must be paired with transparency, data protection, and digital literacy initiatives to realize its full potential. Further, Skepticism significantly moderates the indirect effect via perceived social presence (Index = −0.109, 95% CI [−0.187, −0.042]), but not via trust (Index = −0.040, 95% CI [−0.081, 0.003]), indicating the cognitive pathway is more robust to skepticism—consistent with Privacy Calculus Theory (see Table 9).

#### 4.3.4. Robustness Analysis

Robustness analyses confirm that the inclusion of control variables (age, gender, education, income, prior AI experience, bank type, smartphone proficiency, and region), did not substantively alter the direction, significance, or magnitude of hypothesized relationships (Δβ < 0.02; see Table 10).

**Figure 3:**
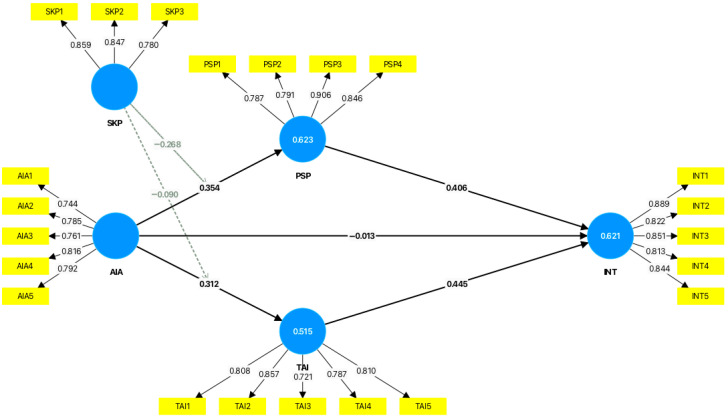
Structural model.

The structural model (see Figure 3) confirms perceived social presence and trust in the AI system as key mediators between AI anthropomorphism and intention to adopt AI-driven banking services. Trust in AI also directly impacts adoption intention, while AI anthropomorphism does not exert a significant direct effect. A key finding is that skepticism negatively moderates both the AIA–PSP and AIA–TAI relationships, indicating that the effectiveness of anthropomorphic design is significantly diminished among highly skeptical users.

## 5. Discussion

This study investigates how AI anthropomorphism shapes customer adoption of AI-driven banking services in Sierra Leone—a fragile economy marked by institutional fragility, emerging digital infrastructure, and deep-seated relational norms in financial service delivery. By integrating the Stimulus–Organism–Response (S-O-R) framework with the Computers Are Social Actors (CASA) paradigm, Institutional Trust Theory, and Privacy Calculus Theory, we analyze direct, mediated (via perceived social presence and trust), and moderated (by skepticism) effects. This study investigates the psychological pathways linking AI anthropomorphism to adoption intention in Sierra Leone. The findings provide associational evidence that challenges the universal applicability of human-like AI design principles and offers context-specific insights that can inform future research and design thinking for AI deployment in fragile economies.

### 5.1. AI Anthropomorphism and Adoption Intention

The strong, positive associations of AI anthropomorphism on perceived social presence and trust in the AI system are consistent with the CASA paradigm, which posits that users automatically apply social heuristics to machines exhibiting human-like cues—even when aware of their artificial nature (Nass & Moon, 2000). This aligns with meta-analytic evidence demonstrating that anthropomorphic design reliably enhances social presence and trust across service contexts (Blut et al., 2021). In Sierra Leone’s interpersonal banking culture where face-to-face rapport remains normative, anthropomorphic features such as empathetic language or a personalized voice help bridge the emotional gap between impersonal technology and human-centered service expectations (T. Li et al., 2025) This finding resonates with recent works of (Bhatnagr et al., 2024; Schrank, 2025), who argues that Anthropomorphic AI features, including perceived intelligence and human-like characteristics, enhance interaction quality and predict continued usage intentions in digital banking services.

Conversely, the non-significant direct effect of AI anthropomorphism on adoption intention (β = −0.013, *p* = 0.760) is arguably the study’s most theoretically significant finding. It starkly contrasts with studies in WEIRD contexts that report consistent direct links (Kumar et al., 2025), where anthropomorphic cues appear to influence behavior directly without requiring full cognitive mediation. The absence of a direct effect in Sierra Leone reveals a critical boundary condition on CASA: in fragile economies marked by institutional fragility and weak data governance, surface-level social heuristics are insufficient to drive adoption. Users do not adopt AI because it ‘feels human’; they require that this humanity be translated into verifiable psychological states—connection (social presence) and assurance (trust). This pattern positions anthropomorphism not as a direct persuasive tool but as a relational amplifier whose behavioral influence is contingent on deeper cognitive-affective processing. The null direct effect, therefore, is not a disconfirmed expectation but evidence that in low-trust environments, design stimuli must be validated through internal appraisal before shaping behavior. Thus, our results challenge the assumed universality of CASA and demonstrate that in low-trust environments, surface-level heuristics must be validated through deeper cognitive appraisal before influencing behavior.

### 5.2. The Dual Mediating Pathways: Social Presence and Trust

The findings robustly confirm perceived social presence and trust in the AI system as essential psychological mediators. Both constructs fully transmit the influence of anthropomorphism to adoption intention. Social presence operates as the initial affective gateway: when users feel they are interacting with a sentient, responsive entity, anxiety around digital transactions diminishes (Vazquez et al., 2023). Trust emerges as the stronger proximal driver, reflecting the high-stakes nature of banking where benevolence and data integrity outweigh mere co-presence. Most significantly, the mediation analysis reveals that AI anthropomorphism is associated with adoption exclusively through these internal states. This positions social presence and trust not as secondary outcomes but as the core transmission mechanisms through which design stimuli become behavioral intent. Given the limited technical schemas available to users in Sierra Leone for evaluating AI, such relational cues act as essential cognitive heuristics. An AI that “listens” and “protects” satisfies deep-seated expectations of financial stewardship, converting anthropomorphic cues into actionable trust.

### 5.3. The Contingent Role of Skepticism

The moderation analysis delivers the study’s most contextually profound insight: skepticism functions as a powerful boundary condition that reconfigures the efficacy of anthropomorphic design. The negative interactions between skepticism and anthropomorphism on both social presence and trust confirm that in environments with weak data governance and historical institutional distrust, users engage in threat appraisal rather than social acceptance. The results further reveal that among low-skepticism users, anthropomorphism strongly enhances social presence and trust; among high-skepticism users, these effects are weakened by 83% and 50%, respectively. Critically, however, the index of moderated mediation indicates that this attenuation significantly disrupts the indirect pathway to adoption only for social presence, whereas the trust pathway remains partially robust. Specifically, under high skepticism, the indirect effect via social presence becomes non-significant (β = 0.035, 95% CI [−0.012, 0.098]), while the indirect effect via trust remains significant albeit reduced (β = 0.099, 95% CI [0.041, 0.172]).

This pattern aligns with Privacy Calculus Theory (Dinev & Hart, 2006), suggesting that skeptical users engage not in social attribution but in threat appraisal, interpreting human-like cues not as benevolent but as potentially manipulative. Consequently, anthropomorphism’s relational benefits are contingent on a foundational level of user trust—a condition that cannot be engineered through interface design alone in contexts marked by systemic institutional distrust. Skepticism in Sierra Leone reflects a contextually rational stance, given uneven digital literacy and emerging regulatory frameworks. Consequently, anthropomorphism’s effectiveness is contingent not merely on design quality but on the readiness of the socio-institutional context to support trust.

## 6. Implications of the Study

### 6.1. Theoretical Implications

This study advances human–AI interaction theory in three interlocking ways.

First, this study advances human–AI interaction theory by establishing institutional fragility as a critical boundary condition for prevailing AI adoption models. First, we reframe AI anthropomorphism not as a universal design heuristic—as often assumed in Western-derived literature—but as a context-contingent stimulus whose efficacy is conditional on socio-institutional infrastructure. While prior research largely situated in WEIRD contexts treats anthropomorphism as a direct catalyst for engagement (J. Wang et al., 2025), our findings demonstrate that in fragile economies, its influence is fully mediated by psychological states (social presence and trust) and severely weakened by skepticism. This challenges the implicit universality of the CASA paradigm (Nass & Moon, 2000), revealing its limits when users lack the institutional framework to interpret social cues as benevolent. In doing so, we extend the S-O-R framework beyond its origins into domains of institutional voids, where stimuli are filtered through risk calculus rather than social heuristics.

Second, the findings suggest a more complex operation of the ‘organism’ in the S-O-R framework than is often depicted in WEIRD-context studies. In Sierra Leone, anthropomorphism appears to simultaneously activate two distinct psychological pathways—an affective-relational one (social presence) and a cognitive-institutional one (trust). This pattern is consistent with the idea of dual-channel appraisal, a hypothesized adaptive response in contexts of institutional fragility. We acknowledge, however, that this dual-channel interpretation is inferential based on our cross-sectional data and would require experimental or process-tracing validation to confirm the cognitive temporality. Future research could experimentally test whether this represents truly parallel processing or a rapid, sequential evaluation.

Third, the findings point to a potential duality in the formation of trust in fragile economies. The strong moderating role of skepticism suggests that trust is not simply granted based on surface-level cues but is actively ‘negotiated’ by the user, who weighs relational signals against perceived institutional risks. In Sierra Leone’s low-trust banking environment, trust is not granted—it is negotiated. Anthropomorphic cues only foster trust when users perceive them as authentic signals of accountability, not as persuasive tactics. This synthesis of Institutional Trust Theory (Zucker, 1986), which posits that in weak-institution settings, trust substitutes for formal safeguards, and Privacy Calculus Theory (Dinev & Hart, 2006), which frames technology adoption as a risk-benefit trade-off, reveals that trust in AI is not merely cognitive or affective, but institutionally embedded. When regulatory oversight is weak, users demand more than likability; they require verifiable integrity. Trustworthy AI systems require comprehensive requirements, including explainability, accountability, robustness, and human oversight to ensure safety and reliability before deployment (Alzubaidi et al., 2023). This concept of a ‘negotiated trust’, where user skepticism acts as a key filter, emerges as a central insight from our exploratory analysis and warrants further investigation.

Fourth, this study directly responds to enduring calls for contextual theorizing in information systems research (Grover & Lyytinen, 2023). By demonstrating that CASA mechanisms appear attenuated under conditions of high skepticism, we highlight the potential epistemic bias of importing Western theories into Global South contexts without adaptation. In post-conflict societies, findings suggest users may not passively accept social cues; rather, they appear to interrogate them through a lens of structural vulnerability. Consequently, AI adoption is not a matter of interface aesthetics but of relational legitimacy—a process requiring transparency, data sovereignty, and co-designed governance (Couldry & Mejias, 2019). This reframing shifts the theoretical focus from user-centered design to contextually accountable design, aligning with recent decolonial critiques of AI ethics that emphasize power, equity, and overlooking African philosophies (van Norren, 2023). This suggests that effective AI deployment in fragile economies requires not just user-centered design but ‘contextually accountable design’ that acknowledges technology is never neutral in settings marked by colonial technological legacies (Wakunuma et al., 2025). Collectively, these contributions move the field beyond universalist assumptions toward a situated, institutionally aware theory of human–AI interaction—one that acknowledges that in fragile economies, technology is never neutral, and design is never enough.

### 6.2. Practical Implications

The practical implications of this study call for a fundamental shift in strategy from a design-centric to a trust-centric approach for deploying AI-driven banking services in fragile economies like Sierra Leone. For financial institutions in fragile economies, the findings suggest that building foundational trust may be a prerequisite for anthropomorphic design to be effective. This points to a potential strategy of prioritizing transparency and reliability in initial AI deployments, with human-like features introduced later to enhance, rather than create, user trust. This is an associational insight that would benefit from experimental validation. The strong moderating effect of skepticism raises the possibility that different user segments may respond differently to anthropomorphic design. Banks could explore this further by conducting A/B testing to see if less skeptical users indeed show a stronger preference for human-like interfaces, while more skeptical users respond better to functional, transparent designs. Our study provides the initial hypothesis for such tests. For AI developers, the findings highlight the importance of context-sensitive design. Allowing users some control over the interface’s social cues (e.g., toggling between a simple, text-based interaction and a more conversational one) could be a way to accommodate varying levels of user skepticism. This design idea is one potential implication of our data. Finally, International development partners may complement technology transfer with investments in localized trust infrastructure—such as independent AI audit mechanisms and contextually adapted consumer protection frameworks. Our associational findings suggest anthropomorphism’s efficacy depends on pre-existing trust conditions; however, longitudinal or experimental validation is needed before translating this insight into policy. Context-sensitive co-development of trust infrastructure alongside technological solutions could offer a more sustainable pathway for AI adoption in fragile economies. Ultimately, this study underscores that in low-trust environments, anthropomorphism is merely an amplifier of trust, not a generator of it; sustainable adoption depends on first addressing the institutional and experiential roots of skepticism through transparency, accountability, and proven integrity.

### 6.3. Research Limitations and Future Research

This study has several limitations. First, the cross-sectional design precludes causal inference regarding temporal dynamics of trust formation; longitudinal or experimental designs are needed to establish directionality and test whether skepticism attenuates with sustained AI exposure. Second, the sample overrepresents urban, digitally literate customers with high education and smartphone ownership, limiting generalizability to rural, unbanked populations central to financial inclusion. These groups may use different access channels (e.g., USSD), rely on community-based trust, and face infrastructural constraints, suggesting the identified psychological pathways may not hold. Future research requires inclusive sampling strategies targeting these populations. Third, despite procedural safeguards and statistical controls, single-source cross-sectional data remain susceptible to common method bias. While triangulated CMB tests suggest minimal distortion, residual bias cannot be ruled out. Fourth, findings reflect Sierra Leone’s specific post-conflict context and may not generalize across fragile economies with different institutional voids or cultural trust schemas. Fifth, anthropomorphism was measured as a perceived attribute based on prior exposure rather than a manipulated stimulus, introducing heterogeneity. While this enhances ecological validity, it precludes causal claims about specific design features. Consequently, our findings address psychological mechanisms linking perceived anthropomorphism to adoption, not design-level interventions. Finally, while cultural adaptation enhanced ecological validity, embedding institutional references in trust items (e.g., ‘Even though data laws are weak here…’) may have primed respondents to link trust directly to institutional fragility, potentially inflating the observed association between skepticism and trust due to shared contextual cues; subsequent inquiry should employ context-neutral items to disentangle construct-specific variance from contextual priming effects.

Future research should (1) use experimental designs to isolate which anthropomorphic cues most effectively activate trust and when they backfire; (2) adopt longitudinal designs tracking trust calibration over time and following service failures; (3) employ experience sampling to capture real-time responses; (4) adopt inclusive sampling via low-bandwidth interfaces (USSD, voice-only) reaching marginalized rural users; (5) integrate behavioral metrics (e.g., usage logs) with surveys to bridge intention–behavior gaps; (6) conduct comparative studies across Global South contexts to distinguish universal from culturally contingent adoption pathways; and (7) future experimental research should employ standardized stimuli to isolate which anthropomorphic cues effectively activate trust or trigger skepticism.

## 7. Conclusions

This study delineates critical boundary conditions for the broader AI adoption literature. We find that in Sierra Leone’s fragile banking context, AI anthropomorphism is associated with adoption only indirectly—through perceived social presence and trust—with no direct effect. This absence of a direct effect is not a null finding but a theoretically significant discovery: it suggests that in low-trust environments, human-like design cannot bypass users’ cognitive-affective appraisal systems. Anthropomorphism functions not as a direct persuasive cue but as a relational amplifier whose behavioral influence is entirely mediated by the psychological states it activates. These findings challenge the assumed universality of human-like AI design, revealing it as a context-contingent strategy that requires foundational trust to succeed. To our knowledge, our study is among the first to examine AI anthropomorphism in banking chatbots within Sub-Saharan Africa’s fragile economies—and the first in Sierra Leone—offering exploratory, context-specific insights into the perceived relational benefits and limitations of human-like AI.

Critically, consumer skepticism sharply weakens these pathways: anthropomorphism’s impact on social presence drops by 83% and on trust by 50% among highly skeptical users. These findings challenge the assumed universality of human-like AI design, revealing it as a context-contingent strategy that requires foundational trust to succeed. To our knowledge, our study is among the first to examine AI anthropomorphism in banking chatbots within Sub-Saharan Africa’s fragile economies—and the first in Sierra Leone— offering exploratory, context-specific insights into the perceived relational benefits and limitations of human-like AI. The findings underscore the need for banks, regulators, and designers to consider the deep-seated role of skepticism in shaping user responses, highlighting that ethical and effective AI deployment in low-trust environments requires more than just good design.

## Figures and Tables

**Table 1:** Respondent Demographics.

Characteristic	Category	*n*	%
Gender	Male	117	42.2
	Female	160	57.8
Age	18–25 years	39	14.1
	26–35 years	70	25.3
	36–45 years	128	46.2
	46+ years	40	14.4
Education Level	Secondary or below	25	9.0
	Diploma/Certificate	73	26.4
	Bachelor’s degree or higher	179	64.6
Employment Status	Employed (formal sector)	137	49.5
	Self-employed/Informal	84	30.3
	Unemployed/Student/Others	56	20.2
Smartphone Ownership	Yes	277	100.0
Bank Type Used	Domestic commercial bank	93	33.6
	Foreign commercial bank	217	78.3
Prior Experience with AI Banking Services	Used chatbot/virtual assistant	189	68.2
	Heard of but never used	88	31.8
Region	Freetown (Western Area)	150	54.2
	Makeni (Northern Province)	44	15.9
	Bo (Southern Province)	45	16.2
	Kenema (Eastern Province)	38	13.7

Source: Authors’ Data.

**Table 2:** Reliability and Validity of Constructs.

Construct	Items	VIF	FL	CA	CR	AVE
AI Anthropomorphism				0.840	0.850	0.608
	AIA1	1.743	0.744			
	AIA2	1.940	0.785			
	AIA3	1.768	0.761			
	AIA4	2.150	0.816			
	AIA5	1.854	0.792			
Perceived Social Presence				0.855	0.878	0.696
	PSP1	1.857	0.787			
	PSP2	1.842	0.791			
	PSP3	2.850	0.906			
	PSP4	1.944	0.846			
Trust in the AI System				0.858	0.877	0.636
	TAI1	2.103	0.808			
	TAI2	2.162	0.857			
	TAI3	1.716	0.721			
	TAI4	1.868	0.787			
	TAI5	1.987	0.81			
Intention to Adopt AI-Driven Banking Services				0.899	0.904	0.713
	INT1	2.974	0.889			
	INT2	2.327	0.822			
	INT3	2.640	0.851			
	INT4	2.076	0.813			
	INT5	2.401	0.844			
Skepticism				0.772	0.774	0.688
	SKP1	1.937	0.859			
	SKP2	1.770	0.847			
	SKP3	1.385	0.780			

Source: Authors’ Data.

**Table 3:** Fornell and Larcker Criterion.

Construct	AIA	INT	PSP	SKP	TAI
AIA	**0.780**				
INT	0.492	**0.844**			
PSP	0.601	0.732	**0.834**		
SKP	0.573	0.712	0.634	**0.829**	
TAI	0.587	0.742	0.750	0.659	**0.798**

Source: Authors’ Data. Note: The bold diagonal elements represent the square root of the Average Variance Extracted (AVE) for each construct. Off-diagonal values represent the inter-construct correlations. For adequate discriminant validity, the diagonal values should exceed the corresponding off-diagonal correlations.

**Table 4:** Heterotrait–Monotrait Ratio.

Construct	AIA	INT	PSP	SKP	TAI
AIA					
INT	0.548				
PSP	0.687	0.817			
SKP	0.707	0.847	0.749		
TAI	0.667	0.816	0.816	0.794	

Source: Authors’ Data.

**Table 5:** Model Performance and Effect Size Estimates (f^2^).

Construct	R^2^	R^2^ Adjusted	Q^2^ Predict	RMSE	MAE	Path	f^2^	Effect Size Interpretation
INT	0.621	0.616	0.49	0.729	0.568	AIA → INT	0	Negligible
PSP	0.623	0.619	0.581	0.666	0.499	AIA → PSP	0.224	Medium–Large
TAI	0.515	0.509	0.497	0.715	0.586	AIA → TAI	0.135	Small–Medium
						PSP → INT	0.173	Medium
						TAI → INT	0.214	Medium–Large

Source: Authors’ Data.

**Table 6:** Direct Effects.

Hypothesis	Path	β	t	*p*	95% CI (Lower, Upper)	Supported?
H1	AIA → PSP	0.354	7.804	<0.001	(0.269, 0.445)	Yes
H2	AIA → TAI	0.312	4.743	<0.001	(0.190, 0.448)	Yes
H3	AIA → INT	−0.013	0.305	0.760	(−0.096, 0.072)	No
H4	PSP → INT	0.406	3.755	<0.001	(0.180, 0.604)	Yes
H5	TAI → INT	0.445	5.538	<0.001	(0.297, 0.611)	Yes

Source: Authors’ Data.

**Table 7:** Mediation Effects.

Hypothesis	Mediation Pathway	Specific Indirect Effect (β)	Bootstrapped 95% CI	t	*p*	Proportion of Total Indirect Effect
H6	AIA → PSP → INT	0.144	[0.062, 0.226]	3.433	0.001	50.9%
H7	AIA → TAI → INT	0.139	[0.068, 0.210]	3.889	<0.001	49.1%
—	Total Indirect Effect	0.283	[0.172, 0.401]	5.127	<0.001	100%
—	Direct Effect (AIA → INT)	−0.013	[−0.096, 0.072]	0.305	0.760	—
—	Total Effect	0.270	[0.168, 0.379]	4.984	<0.001	—

Source: Authors’ Data.

**Table 8:** Moderation Effects.

Hypothesis	Interaction Path	β	t	*p*	Supported?
H8	SKP × AIA → PSP	−0.268	5.657	0.000	Yes
H9	SKP × AIA → TAI	−0.090	3.231	0.001	Yes

Source: Authors’ Data.

**Table 9:** Conditional Indirect Effects and Index of Moderated Mediation.

Mediator	Skepticism Level	Conditional Indirect Effect (β)	Bootstrapped 95% CI
PSP	Low (−1 SD)	0.253	[0.162, 0.358]
PSP	High (+1 SD)	0.035	[−0.012, 0.098]
Index of moderated mediation	—	−0.109	[−0.187, −0.042]
TAI	Low (−1 SD)	0.179	[0.112, 0.264]
TAI	High (+1 SD)	0.099	[0.041, 0.172]
Index of moderated mediation	—	−0.04	[−0.081, 0.003]

Source: Authors’ Data.

**Table 10:** Comparison of Key Path Coefficients: Models With vs. Without Control Variables.

Path	Full Model (With Controls)	Reduced Model (Without Controls)	Δβ	*p*-Value (Difference)
AIA → PSP	0.354 ***	0.361 ***	0.007	0.682
AIA → TAI	0.312 ***	0.325 ***	0.013	0.541
AIA → INT	−0.013	−0.009	0.004	0.891
PSP → INT	0.406 ***	0.398 ***	−0.008	0.723
TAI → INT	0.445 ***	0.451 ***	0.006	0.814
SKP × AIA → PSP	−0.268 ***	−0.274 ***	−0.006	0.765
SKP × AIA → TAI	−0.090 ***	−0.095 ***	−0.005	0.832
R^2^ (INT)	0.621	0.618	−0.003	—
R^2^ (PSP)	0.623	0.615	−0.008	—
R^2^ (TAI)	0.515	0.509	−0.006	—

Note: *** *p* < 0.001. Δβ = absolute difference in standardized coefficients between models. *p*-values for differences derived from 5000 bootstrap resamples comparing coefficient distributions.

## Data Availability

The data used to support the findings of this study are available from the corresponding author upon request.

## Appendix A. Measurement Scales

**Variable**	**Items**	**Source**
AI Anthropomorphism	The AI assistant seems to have human-like qualities.	(Bartneck et al., 2009)
	The way the AI speaks sounds warm and friendly, like a person.	
	The AI assistant appears to understand my emotions.	
	The AI has a personality that feels real.	
	The AI explains decisions in plain language you understand.	
Perceived Social Presence	I feel like I am interacting with a real person.	(Gefen & Straub, 2004)
	The AI assistant gives me a sense of being “together” during our interaction.	
	I feel that the AI is attentive to what I say.	
	The interaction feels socially engaging, not mechanical.	
Trust in the AI System	Even though data laws are weak here, this AI tries to protect my information.	(Chowdhury et al., 2022)
	The AI is competent in handling my banking needs.	
	I feel confident that the AI will act in my best interest.	
	The AI is honest and truthful in its responses.	
	I can rely on the AI to perform as expected.	
Intention to Adopt AI-Driven Banking Services	I intend to use AI-driven banking services in the future.	(Liu et al., 2022)
	I plan to rely on AI for my banking inquiries.	
	I would recommend using AI banking services to others.	
	I will continue using AI banking services if available.	
	I am willing to adopt new AI features offered by my bank.	
Skepticism Toward AI	I doubt that an AI can truly understand my financial needs.	(Zhang et al., 2016)
	I am suspicious about how my data is used by AI banking systems.	
	I believe AI banking services are more about corporate control than customer benefit.	
